# Activation of the rostromedial prefrontal cortex during the experience of positive emotion in the context of esthetic experience. An fNIRS study

**DOI:** 10.3389/fnhum.2013.00879

**Published:** 2013-12-20

**Authors:** Ute Kreplin, Stephen H. Fairclough

**Affiliations:** School of Natural Science and Psychology, Liverpool John Moores UniversityLiverpool, UK

**Keywords:** fNIRS, BA10, emotion, esthetics, prefrontal cortex

## Abstract

The contemplation of visual art requires attention to be directed to external stimulus properties and internally generated thoughts. It has been proposed that the medial rostral prefrontal cortex (rPFC; BA10) plays a role in the maintenance of attention on external stimuli whereas the lateral area of the rPFC is associated with the preservation of attention on internal cognitions. An alternative hypothesis associates activation of medial rPFC with internal cognitions related to the self during emotion regulation. The aim of the current study was to differentiate activation within rPFC using functional near infrared spectroscopy (fNIRS) during the viewing of visual art selected to induce positive and negative valence, which were viewed under two conditions: (1) emotional introspection and (2) external object identification. Thirty participants (15 female) were recruited. Sixteen pre-rated images that represented either positive or negative valence were selected from an existing database of visual art. In one condition, participants were directed to engage in emotional introspection during picture viewing. The second condition involved a spot-the-difference task where participants compared two almost identical images, a viewing strategy that directed attention to external properties of the stimuli. The analysis revealed a significant increase of oxygenated blood in the medial rPFC during viewing of positive images compared to negative images. *This finding suggests that the rPFC is involved during positive evaluations of visual art that may be related to judgment of pleasantness or attraction. The fNIRS data revealed no significant main effect between the two viewing conditions, which seemed to indicate that the emotional impact of the stimuli remained unaffected by the two viewing conditions.*

## Introduction

The experience of viewing art is influenced by a modulation of attentional focus between external features of the stimuli and internal feelings/thoughts. Internal cognitive processes such as object recognition, memory recall and mental imagery facilitate content recognition during the viewing of visual art (Fairhall and Ishai, 2008). Recognition of familiar content evokes a pattern of activation in multiple extrastriate ventral and dorsal regions, the hippocampus, intra parietal sulcus and inferior frontal gyrus (Ishai et al., 2007; Fairhall and Ishai, 2008; Nadal et al., 2008; Nadal and Pearce, 2011). According to a MEG time frequency analysis, a peak of acitivity around 170 ms has been related to the commencement of coding for object identity and transformation of sensory code to cognitive processing has been associated with a peak of activity at approx. 170 ms during the observation of visual art (Munar et al., 2011). This process of feature extraction from visual art and the generation of associated thought and feelings have a distinct temporal window.

The rostral prefrontal cortex (rPFC) may be an important site of activity during the processing of art; this region has been associated with higher order cognitive processes such as prospective memory (Volle et al., 2010; McDaniel et al., 2013), emotional regulation strategies (Amting et al., 2010; Viviani et al., 2010; Campbell-Sills et al., 2011) and sustained attention (Van Veen and Carter, 2006; Ernst et al., 2012). However, there is little consensus regarding the functional specificity and cytoarchitecture of the prefrontal cortex (PFC), particularly the rostral area of the PFC. Ramnani and Owen (2004) suggested that the rPFC is activated when the outcomes of two or more separate cognitive operations require integration in the pursuit of a higher behavioral goal. Other accounts emphasized the involvement of medial rPFC during the processing of self-related information (Seitz et al., 2009; Denny et al., 2012).

The gateway hypothesis (Burgess et al., 2003, 2007) was developed to connect activation in the rPFC to higher-order self-referential processing and the evaluation of internally generated information. According to this model, rostromedial areas of the PFC (medial BA10) are implicated in the maintenance of attention towards external stimuli whereas activation of the rostrolateral areas (lateral BA10) are associated with the preservation of attention on internal cognitions; this functional differentiation is proposed to act as a gateway between the direction of attention towards external and internal stimuli (Burgess et al., 2007). The central proposal of the model is that the rPFC is part of a system that allows conflicts to be resolved during ambiguous situations (where information activating relevant schemata has low triggering input) or increases activation of schemata in accordance with higher-level goal representations (Cupchik et al., 2009; Volle et al., 2010; Henseler et al., 2011). Evidence for this functional differentiation has been observed during the comparison of shapes (Henseler et al., 2011), letters (Gilbert et al., 2005; Benoit et al., 2012) and the identification of features between two different stimuli such as texture or aspects of geometric shapes (Volle et al., 2010).

The general function of the rPFC as described by the gateway hypothesis is twofold. This area enables the activation of schemata in a situation where no schema is sufficiently triggered by incoming stimuli, e.g., if the stimulus is entirely novel and cannot be associated with existing information. Secondly, the rPFC enables attentional bias when many schema are simultaneously activated (e.g., if a situation is very difficult or complex) or if there are a multitude of possible established outcomes without an obvious advantage to one of them (Burgess et al., 2005). The rPFC plays a key role in the goal-directed co-ordination of stimulus-independent and stimulus-orientated cognitions in situations where established patterns of behavior are insufficient. Stimulus-independent cognitions include introspection or creative thoughts which are neither provoked by nor directed toward external stimuli. Stimulus-oriented cognitions represent the opposite category, being provoked and oriented towards sensory input. The rPFC would be typically activated in situations that are novel or where a specific demand for it has been determined (e.g., “I must pay special attention to…”, “I must think about…”; Burgess et al., 2003, 2005, 2007; Gilbert et al., 2005; Volle et al., 2010; Benoit et al., 2012). The contemplation of visual art requires a shift from stimulus-dependent processing to those stimulus-independent processes that permits an assessment of stimuli as being esthetically pleasing or not. It may be hypothesized that attention is directed to external properties of the stimulus (identification of physical properties within the painting), the reinvestment of attention onto internally generated thoughts (what does the artist want to say with this painting), and the reinvestment of attention onto subjective self or personal entity (what does the painting mean to me). It is plausible that the rPFC is involved in object identification as well as directing attention to those self-referential states that are relevant to esthetic appreciation, but the roles of medial and lateral areas of the rPFC during the contemplation of visual art as defined by the gateway hypothesis remains unclear.

There is evidence connecting activity in the rPFC to aspects of cognition that are implicit within emotional processing, e.g., attention to emotion, emotion regulation, appraisal or interpretation of emotion. For instance, Phan et al. (2002) reported strong connections between the PFC (BA9/10) and the anterior cingulate cortex (ACC) and suggested that both areas of the PFC could serve as top-down modulators of intense emotional responses (see also Amting et al., 2010; Holroyd and Yeung, 2012). Evidence for this interpretation stems from human lesion studies where damage to the PFC leads to socially inappropriate expressions of emotions and impairment in making advantageous personally relevant decisions suggesting a lack of awareness/comprehension of emotionally “loaded” situations (Damasio, 1999; Leopold et al., 2012; Maier and di Pellegrino, 2012). Similarly, activation of the rPFC was related in a linear fashion with an emotion induction task that required different degrees of self-monitoring (identifying with the feelings/emotions depicted in a picture compared to just viewing a picture) (Herrmann et al., 2003; see Denny et al., 2012 for a review).

Studies relating activity in the rPFC to the process of emotional regulation present an alternative to the gateway hypothesis. The regulation of emotions has been associated with activation of the rPFC during the up- and down regulation of emotions (Mitchell, 2011). Furthermore medial BA10 has been linked to activity related to the subjective self or personal entity (Amting et al., 2010; Denny et al., 2012). Seitz et al. (2009) suggested that nodes in the medial PFC participate in early processing of sensory information and mediate the value judgment of the stimulus by assessing self-relevant meaning to the sensations. An esthetic experience, or more specifically the contemplation of visual art, is a highly subjective process. It is therefore important that the experiences are self-referential, particularly if the viewer is not trained in an art-related subject and bases his/her evaluation of visual art largely on personal experiences. Evidence supporting this interpretation and the involvement of the medial rPFC was provided by Vessel et al. (2012) who reported an increase of activation in the medial rPFC for those paintings subjectively judged as most esthetically moving. This activation of the medial rPFC was specific to the assessment of esthetic pleasure. This study suggested that a highly-subjective emotional connection to visual art is important to the esthetic experiences and activation in the rPFC was associated with this type of experience. Alternatively, the involvement of the lateral rPFC during esthetic experiences was reported by Cupchik et al. (2009) who investigated how cognitive control/perceptual facilitation and the experience of emotion contributed to esthetic perception. Participants in this study were instructed to view a painting from either a pragmatic everyday viewpoint or from an esthetic viewpoint. Pragmatic viewing was associated with activation of the fusiform gyrus and areas related to object recognition whereas the esthetic viewing condition activated the insula and left lateral rPFC (BA10). The authors interpreted activation of the latter in terms of stimulus-independent thought related to the contemplation of visual art.

The aim of the current study was to differentiate activation within the rPFC using functional near infrared spectroscopy (fNIRS) during the viewing of visual art selected to induce positive and negative mood. Both categories of image were viewed under two conditions (emotional introspection and external object identification) designed to draw attention to stimulus-independent and stimulus-dependent features respectively. Images were viewed for 60 s overall and split into three viewing periods (early, middle and late) for the analysis. The extended viewing time was chosen because temporal differences have been observed during activation of the PFC during emotional experiences measured by fNIRS, where an overshoot of activation into later periods was reported (León-Carrion et al., 2008), and during the experience of art (Munar et al., 2011). Our primary hypothesis was that emotional introspection would activate lateral areas of the rPFC whereas viewing the image with an emphasis on external object identification would selectively activate the medial rPFC. Our alternative hypothesis was that emotional introspection would activate the medial rPFC as predicted by the studies conducted by Seitz et al. (2009) and Vessel et al. (2012).

## Methods

### The survey

Standardized visual materials, such as the International Affective Picture System (IAPS; Lang et al., 1993) are often used to provoke emotional experience under controlled conditions. The purpose of the survey exercise was to create a database of esthetic images with known psychological properties for experimental purposes. Sixty-three paintings including both representational and abstract images were selected from online sources; all artists had sold or exhibited their work and agreed to allow their work to be used in the survey exercise. Participants were asked to rate each image on six components represented by 9-point Likert scales. Three components represented cognitive components, these scales were labeled: complexity (how complex was this image?), comprehension (do you understand this image?) and novelty (how novel or unusual was the image?). The remaining three components represented emotional factors including: valence (is the image positive or negative?), activation (do you find the image stimulating) and attractiveness (do you find the image attractive or repellent?). The cognitive components were derived from Silvia’s (2008) analysis of interest whilst emotional components were based upon the Self-Assessment Mannikin (Lang et al., 1993).

The survey was made available online and 1043 participants (63% female) with a mean age of 33 years. (s.d. 14.24) provided ratings for the images. The resulting database was used to select images rated as positive/negative valence for the experimental study.

### Experimental study

#### Participants

Thirty right handed participants (15 female) were recruited from an undergraduate population. Participants had a mean age 22 years. (s.d. = 3.26 years) with no formal training in an art-related subject and no history of neurological disorder. Participants were informed about the procedure and operating mode of the fNIRS prior to providing written consent. All procedures were approved by the University Research Ethics Committee prior to data collection.

#### Experimental task

The stimuli were projected onto a white wall in front of the participants using E-prime 2.0 (PST Inc.); the image dimensions were 2040 × 786 pixels. The viewing distance to the screen was approx. 1.90 m. Each stimulus was presented for 60 s and preceded by a 60 s baseline consisting of a light grey screen with a fixation *. Following image presentation, participants were asked to provide ratings for valence and complexity on the scales used in the survey; ratings were recorded in e-prime via a keypad. The tasks were designed to induce different demands on attending to external properties of the stimulus (the spot-the-difference (SD) task) and to internal cognitions (the emotional introspection (EI) task).

**Figure 1:**
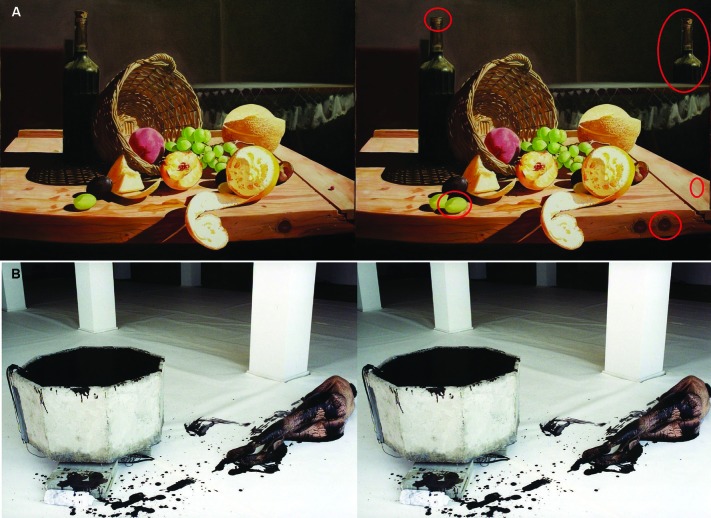
**(A)** Pictures used in the positive SD task (“Best Abstract” © Adrian Borda). The right image has slight differences to the left image, differences are circled in red. **(B)** Two identical images used during negative EI (Monika Weiss “Elytron” 2003, self-shot photography, performance, installation, sculpture and video. Courtesy the artist and Chelsea Art Museum, New York). Participants were asked to think about how the images made them feel.

We closely considered the prerequisites stated by Burgess et al. (2005) that a task requires to draw attention to external stimulus processing when designing the SD task. According to Burgess et al. (2005) an external attentional task requires: (1) that the information to be processed is currently available (i.e., present in the sensory environment); (2) that the attention is directed to external stimuli or stimulus features; and (3) that the operations involved prior to responding are relatively automatic or well-learnt. To elicit attending to external stimuli features during the SD task, all images were duplicated to form a pair and between three and six aspects of the image on the right were modified using PaintShopPro (Figure 1A). Instructions in the SD condition stated: “If you see “SD” on the screen you will be asked to spot the differences between two images. You will be able to see the answers after the image”. Following the ratings in the SD task participants were provided with the same picture but with the differences highlighted with a red circle. All images were used in the SD task and the emotion induction task. Participants were prompted with SD or E before the image appeared as indicator which task to perform next during testing.

The EI condition was designed to initiate internal processes with respect to: (1) the information attended to is being processed internally; (2) that this information is self-generated or comes from a previously witnessed episode; and (3) that the response to be made are triggered by these internal representations. Instructions were as follows: “If you see an “E” on the screen you are asked to think about how the artwork makes you feel, i.e., what emotions does it trigger in you? Does it make you feel sad/happy/angry etc.? Does it remind you of an emotional event you have experienced in the past? Don’t worry about the message the artist tried to bring across, just think about how the image makes YOU feel or if it makes you think of something that you found emotional”. Participants were asked to write, using pen and paper, brief notes about these associations following the picture presentation. Participants were not asked to hand these notes to the experimenter and took them away upon completion of testing to assure confidentiality. The images were presented as identical pairs during the EI task to ensure consistency with the SD viewing condition (Figure 1B). A practice trial was completed for each condition, and the opportunity to ask questions was given to insure the instructions were fully understood before the start of the experiment. The presentation of positive/negative images and EI/SD was randomized.

##### Stimuli

Sixteen images (8 in a positive valence category and 8 in a negative valence category) were selected according to ratings of valence and complexity obtained during the survey (see section The Survey). Mean ratings for positive images were 2.57 (+/− 1.27) and negative images 7.17 (+/− 1.3), whilst subjective ratings of image complexity were constant between positive and negative images (mean 4.58 +/− 2.22 and 4.07 +/− 2.14 respectively).

#### Procedure

Participants were informed about the nature of the study upon arrival and provided written consent before the fNIRS device was fitted. Participants were informed about the study tasks through written instructions projected onto the wall in front of them. Participants had the opportunity to ask questions before and during a practice trial for each task that was shown after the instructions. The experimental protocol began after the experimenter was satisfied that the participant understood the experimental tasks. Participants were thanked for their participation following the experiment and compensated with a £10 voucher for their time.

#### fNIRS data collection

**Figure 2:**
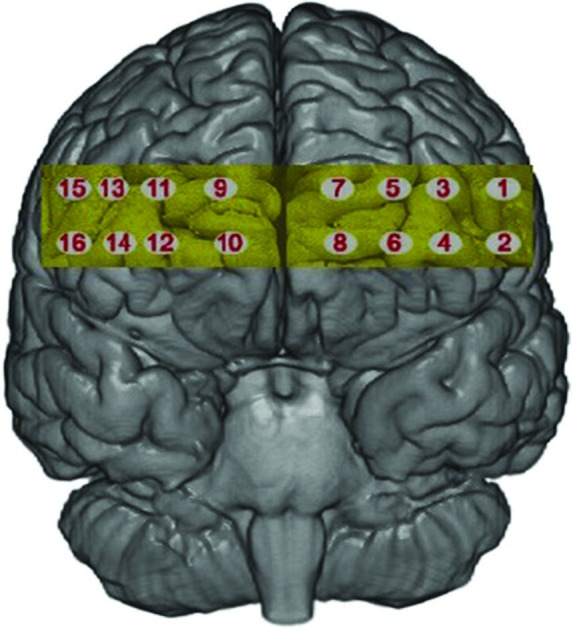
**The 16 voxels of the fNIRS probe located over the rPFC**.

fNIRS was recorded using fNIR Imager1000 and COBI data collection suit (Biopac System Inc.) and is described in detail elsewhere (Ayaz et al., 2010). The system has a temporal resolution of about 500 ms for one complete data acquisition cycle (about 2 Hz). The 16 channel probe was placed on the forehead aligned to Fp1 and Fp2 of the international 10–20 system, and rotated so that Fpz corresponded to the midpoint of the probe (Ayaz et al., 2006; Figure 2). Areas underlying the 16 voxels are right and left superior and inferior frontal gyrii (BA10 and BA46). Cognitive and emotional functions associated with BA10 and BA46 have been explored using fNIRS application similar to the device used in this work (Plichta et al., 2006; Izzetoglu et al., 2007; León-Carrion et al., 2008).

fNIRS data were analyzed offline using fNIRS-Soft (Ayaz et al., 2010). Raw data was subjected to a Sliding-window Motion Artefact Rejection (SMAR) algorithm to remove motion artefacts and saturated channels (Ayaz et al., 2010). Oxygenated haemoglobin (HbO) and deoxygenated haemoglobin (HHB) were calculated using the modified Beer-Lambert Law. A finite impulse response linear phase low-pass filter, with order 20 and cut-off frequency of 0.1 Hz was applied to attenuate high frequency noise, respiration and cardiac effects (Izzetoglu et al., 2007; Ayaz et al., 2010). Sixteen segments with durations of 60 s were extracted using synchronization markers. Segments were averaged according to condition (Positive EI, Negative EI, Positive SD, Negative SD).

There is currently no strong consensus in the literature regarding the optimal feature of brain activation that can be derived from fNIRS data. Research investigating emotional processes using fNIRS has reported significant changes in the PFC for HbO alone (León-Carrion et al., 2008), for HHb alone (Ernst et al., 2012) or for both HbO and HHb (Glotzbach et al., 2011). It has been argued that HHb is sensitive to local haemodynamic changes, less prone to influences from psychophysiological noise, such as breathing or heart rate and has a close association with the blood oxygenation dependent (BOLD) signal obtained from fMRI. However, HbO is the parameter which is less sensitive to variation in probe placement due to head size and shape because HbO activation is more global compared to HHb activation (Wobst et al., 2001; Hoshi, 2005; Plichta et al., 2006). We decided to calculate a compound score for oxygenation (Oxy = HbO – HHb) in order to capture both measures whilst controlling for changes in blood volume (Ayaz et al., 2010).

## Results

Individual differences in the subjective evaluation of visual art have been highlighted as a problem in previous studies (e.g., Cupchik et al., 2009; Vessel et al., 2012). Our participants were exposed to four groups of images (EI/positive, EI/negative, SD/positive, SD/negative), each of which contained four individual images. In order to control for individual differences, we selected only those three images from the sample of four that were most representative of their group designation in order to create the best representation of that image category for each participant, i.e., the three images in the EI/negative valence group with maximum scores for valence (rated on a 9-point Likert scale; 1 = positive, 9 = negative) were assumed to represent the best example of EI/negative valence for that particular person.

We conducted a manipulation check of the subjective valence ratings to assess differences between positive and negative images under the two viewing conditions. A 2 (condition) × 2 (valence) analysis of variance (ANOVA) was conducted on subjective ratings from three images in each valence/viewing condition. A significant main effect for valence (*F*(1,29) = 720.50, *p* < 0.01, *η*^2^ = 0.96), and an interaction between condition and valence (*F*(1,29) = 5.06, *p* < 0.03, *η*^2^ = .014) was found. Negative images (mean 7.17, +/− 1.07) were rated as significantly more negative than positive images (mean 2.57, +/− 0.85). Post-hoc analysis showed no significant difference between the EI and SD condition for negative images. For positive images a marginal trend was identified with images in the EI (mean 2.57, +/− 0.85) condition being rated as more positively than those viewed in the SD condition (mean 2.98, +/− 0.78, *p* < .06).

**Figure 3:**
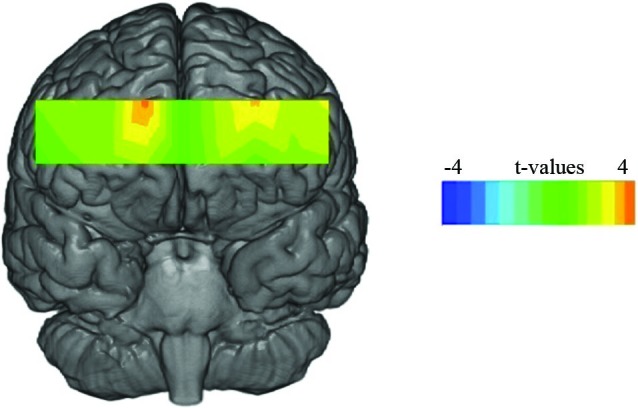
**Statistical maps showing *t*-values for oxygenated blood for the main effect of valence for the 60 s period**.

We separated 60 s of data into three time epochs, early, middle and late, each consisting of 20 s of data. Separate 3 (time) × 2 (condition) × 2 (valence) multivariate analysis of variance (MANOVA) was conducted for each voxel for the compound score oxygenation (Oxy), Greenhouse-Geisser corrections were applied to violations of sphericity. Outliers above and below three standard deviations were excluded. Results yielded a significant main effect for valence at voxel 3 (*F*(1,27) = 5.24, *p* < 0.03, *η*^2^ = 0.16), voxel 5 (Figure 2 for voxel location) (*F*(1,28) = 8.74, *p* < 0.001, *η*^2^ = 0.23) and voxel 9 (*F*(1,26) = 4.32, *p* < 0.04, *η*^2^ = 0.14), all showing greater Oxy for positive images. This effect is illustrated in Figure 3 where heat maps were generated across the rPFC region based on the 16 voxels shown in Figure 2.

## Discussion

The subjective self-report data supported the face validity of the valence manipulation, i.e., positive and negative images were rated appropriately by participants. Our analysis indicated that activation of medial BA10 (as defined by an increase of Oxy) was enhanced during the viewing of visual art that induced positive emotions compared to those paintings that provoked negative emotion (Figure 3). The fNIRS data showed no main effect for viewing condition, which indicated that activation of the medial rPFC was unaffected by the two different viewing conditions.

The increase of activation in response to positive stimuli was unexpected but is not without precedence in the literature (Ernst et al., 2012). It is suggested that positive images activated the medial rPFC because esthetic pleasantness was associated with this category of picture and this effect was unaffected by viewing condition. Previous studies have reported that activation in the medial rPFC is positively correlated with esthetic evaluation (Vartanian and Goel, 2004; Di Dio and Gallese, 2009; Ishizu and Zeki, 2011). When Vessel et al. (2012) found greater activation in the medial rPFC for esthetically pleasing images, they speculated that intense esthetic experiences had high personal relevance, which created a heightened integration of external (sensory/somatic) sensations and internal (evaluative/emotional) states, as the individual experienced an emotional connection to the art. In support of this position, Seitz et al. (2009) suggested that the medial PFC is involved in the attribution of self-relevant, immediate and intuitive meaning. Therefore increased activation of the medial rPFC in the current study may have been the result of self-monitoring of positive emotions or pleasantness judgements during the contemplation of art.

The medial area of the PFC has been implicated in emotional processing in studies with a primary focus on emotion induction (Gray et al., 2002; Glotzbach et al., 2011; Euston et al., 2012; Lindquist and Feldman-Barrett, 2012). Increased prefrontal activation and medial areas in particular, have been associated with self-regulatory strategies designed to minimize negative affect in fMRI studies (for a review see Mitchell, 2011). However, studies investigating self-regulatory strategies during emotional experiences have a tendency to focus on negative emotions such as fear (Glotzbach et al., 2011), anger, sadness, or disgust (Lindquist and Feldman-Barrett, 2012). Herrmann et al. (2003) used positive and negative stimuli to investigate the change of Oxy in the PFC using fNIRS during two types of emotional induction, one with a higher and one with a lower self-monitoring component. The task with the high self-monitoring component resulted in higher levels of oxygenated blood in the medial rPFC regardless of valence. *This study suggests that the rPFC is highly sensitive to emotional induction tasks where an element of self-monitoring is implicated. However, we found activation only for positive images, regardless of viewing condition, and it is unclear why negative emotions would not have activated the rPFC unless the emotional response was specific to esthetic pleasure or the beauty of the picture.*

The gateway hypothesis proposed a functional differentiation between activation for stimulus-dependent and stimulus-independent cognitions in the rPFC (Burgess et al., 2003, 2007). However, the current study found no evidence to support this position because the two viewing conditions had no significant effect on activation in the rPFC. It could be argued that the SD task may not have been suitable to draw attention toward external properties of the stimulus. However the same task was successful in eliciting the functional differentiation in rPFC activation described by the gateway hypothesis in a previous study (Volle et al., 2010). Our stimuli consisted of complex visual art which contrasts with previous research that used simple geometric patterns (Volle et al., 2010) or shapes (Henseler et al., 2011) to demonstrate the effect described by the gateway hypothesis. The increased complexity of our stimuli may account for the absence of any effect on rPFC activation.

It could be argued that the EI task did not conform to the prerequisites set out by Burgess et al. (2005) for internally generated thoughts because the stimulus was present at all times. To invoke self-referential thoughts, participants were instructed to think about how the images made them feel, how they felt connected to them and what images or memories it provoked in them. The presence of the image throughout the introspective task may have confounded the result but we believe that the EI task used in the current study was ecologically valid with respect to real-life behavior in gallery spaces. Previous findings have reported activation of lateral areas during the contemplation of visual art, and attributed this to a focus on internally generated thought related to the artwork (Cupchik et al., 2009). However, these authors did not manipulate their viewing conditions to investigate the gateway hypothesis during the contemplation of art, but rather used the gateway hypothesis as a possible explanation of increased activity in the lateral rPFC. Nonetheless, future investigation may study the gateway hypothesis during the contemplation of visual art using a paradigm where the stimulus is not present during the EI task.

Future studies investigating the gateway hypothesis may benefit from an emotionally neutral condition during the contemplation visual art and the inclusion of a scale asking for subjective experiences of esthetic pleasantness or beauty. Questions remaining unanswered regarding the absence of rPFC activation during negative emotions and the interaction between a self-relevant and other-relevant focus during the contemplation of art. Future research may consider whether pictures with negative valence can be esthetically pleasing and how this is related to rPFC activation would be of benefit to neuroesthetics.

Our results suggested that emotional processing took precedence over differing viewing instruction during visual contemplation of art. Emotional salience may have been brought to the forefront because of participants search for personal meaning of the art images. Thus participants will have drawn onto their own evaluations and personal association of the art to form a subjective judgement about their value.

## Conflict of interest statement

The authors declare that the research was conducted in the absence of any commercial or financial relationships that could be construed as a potential conflict of interest.
